# Dilated cardiomyopathy caused by a pathogenic nucleotide variant in *RBM20* in an Iranian family

**DOI:** 10.1186/s12920-022-01262-4

**Published:** 2022-05-08

**Authors:** Mahshid Malakootian, Mahrokh Bagheri Moghaddam, Samira Kalayinia, Melody Farrashi, Majid Maleki, Parham Sadeghipour, Ahmad Amin

**Affiliations:** 1 Cardiogenetics Research Center, Rajaie Cardiovascular Medical and Research Center, Iran University of Medical Sciences, Tehran, Iran; 2 Echocardiography Research Center, Rajaie Cardiovascular Medical and Research Center, Iran University of Medical Sciences, Tehran, Iran; 3 Cardiovascular Intervention Research Center, Rajaie Cardiovascular Medical and Research Center, Iran University of Medical Sciences, Tehran, Iran

**Keywords:** DCM, RBM20, Pathogenic variant, Familial

## Abstract

**Introduction:**

Dilated cardiomyopathy (DCM) is characterized by the dilation and impaired contraction of 1 or both ventricles and can be caused by a variety of disorders. Up to 50% of idiopathic DCM cases have heritable familial diseases, and the clinical screening of family members is recommended. Identifying a genetic cause that can explain the DCM risk in the family can help with better screening planning and clinical decision-making. Whole-exome sequencing (WES) has aided significantly in the detection of causative genes in many genetically heterogeneous diseases. In the present study, we applied WES to identify the causative genetic variant in a family with heritable DCM.

**Methods:**

WES was applied to identify genetic variants on a 26-year-old man as the proband of a family with DCM. Subsequently, Sanger sequencing was performed to confirm the variant in the patient and all the available affected and unaffected family members. The pathogenicity of the variant was evaluated through co-segregation analysis in the family and employment of in silico predictive software.

**Results:**

WES demonstrated the missense pathogenic heterozygous nucleotide variant, c.1907G > A, (p.Arg636His, rs267607004, NM_0011343), in exon 9 of the *RBM20* gene in the proband. The variant was co-segregated in all the affected family members in a heterozygous form and the unaffected family members. The in silico analysis confirmed the variant as pathogenic.

**Conclusion:**

Pathogenic *RBM20* nucleotide variants are associated with arrhythmogenic DCM. We believe that our report is the first to show an *RBM20* variant in Iranian descent associated with DCM.

**Supplementary Information:**

The online version contains supplementary material available at 10.1186/s12920-022-01262-4.

## Introduction

Dilated cardiomyopathy (DCM), a myocardial disorder, is characterized by enlarged cardiac ventricles and impaired systolic function, leading to heart failure and premature death [1, 2]. DCM affects 1 in 250–500 people in the general population and is one of the most common forms of inherited cardiomyopathies [3, 4]

DCM phenotypes are determined by genetic and nongenetic causes [5]. Nongenetic forms of DCM may occur with hypertension, valvular heart disease, viral/inflammatory myocarditis, extravagant alcohol consumption, illicit substance use, toxins, and metabolic diseases [6–9]. Genetic variations play a prominent role in the pathogenesis of DCM. Currently, laboratories in the United States and Europe offer different panels ranging from 30 to more than 150 DCM-related genes; nonetheless, most of the genes are only anecdotally associated with the disease or have a putative link on the basis of biological connections with known genes [10, 11]. Moreover, pathogenic gene variants can be determined in 25 to 40% and 10 to 25% of familial and sporadic DCM cases, respectively [6, 12, 13]. In this regard, familial DCM primarily demonstrates an autosomal dominant inheritance pattern, in which a single pathogenic mutation can cause the disease [14, 15]. Autosomal recessive and X-linked inheritances have been shown as well [13].

Whole-exome sequencing (WES) is a viable and powerful technique that beyond traditional candidate gene and locus-mapping methods provides an efficient approach to find the genetic cause of heterogeneous diseases [16, 17] like DCM [18].

In this study, WES was performed on an Iranian family with an autosomal dominant inheritance pattern of DCM.

## Methods

### Subjects

Clinical data were acquired via a thorough cardiac assessment of each individual in the Outpatient Heart Failure Clinic of Rajaie Cardiovascular Medical and Research Center. The diagnosis of DCM was made according to the diagnostic criteria of the European Society of Cardiology (ESC) Working Group on Myocardial and Pericardial Diseases [9]. Cardiac magnetic resonance images and echocardiography and electrocardiography data, as well as blood samples, were obtained.

The study was approved by the Ethics Committee of Rajaie Cardiovascular Medical and Research Center (IR.RHC.REC.1399.078) and was conducted in accordance with the Helsinki Declaration. The proband and 9 of his family members (affected and unaffected) were enrolled in the study.

### DNA extraction and WES

Genomic DNA was isolated from 200 µL of peripheral blood samples from the proband and all the available family members in the pedigree by using a DNA extraction kit (DNPTM Kit, Iran).

WES was done with the SOLIDv4 platform (SureSelect X kit, Macrogen South Korea) following the manufacturer’s instructions. Sequences obtained from WES were aligned to the GRCh37/hg19 human reference genome, and the WES-identified variants were filtered. Briefly, filtering was performed as follow: variants were filtered due to minor allele frequency (MAF) of 1000 Genomes, ExAc, gnomAD, esp6500, Greater Middle East, and Iranome databases, respectively. Then bioinformatics software platforms including Combined Annotation Dependent Depletion (CADD Phred > 15) (https://cadd.gs.washington.edu/), PolyPhen-2 (score = 0–0.15: Benign; score = 0.15–0.85: Possibly damaging; score = 0.85–1:Probably damaging) (http://genetics.bwh.harvard.edu/pph2/), SIFT (score ≤ 0.05: Deleterious; score > 0.05: Tolerable) (https://sift.bii.a-star.edu.sg/), PROVEAN (score ≤ -2.5: Deleterious; score > -2.5: Natural.) (http://provean.jcvi.org/index.php), and MutationTaster (http://www.mutationtaster.org/), were employed to predict the variant’s pathogenicity. The identified variant was classified (graded) in accordance with the American College of Medical Genetics and Genomics (ACMG) guidelines.^5^

### Polymerase chain reaction (PCR), primer design, and sanger sequencing

The confirmation of the putative pathogenic variant detected by WES was carried out via Sanger sequencing with a 3500 Genetic Analyzer (Applied Biosystems, USA) in the proband and all the available family members. Briefly, specific primers were designed to amplify a 940-bp fragment encompassing the candidate pathogenic variant in *RBM20* with the following sequences: forward primer: 5'-TGTGTGGTTCTGTAGAGTTGGG-3' and reverse primer: 5'-CCTAGCGCATAGTAAATAGCCAG -3'.

The cycling conditions for amplifying the region were as follows: initial denaturation at 94 °C for 5 min, followed by 30 cycles of 94 °C for 30 s, 63 °C for 30 s, and 72 °C for 30 s, with a final extension at 72 °C for 10 min. Then, the forward primer was utilized to sequence the part of interest.

### Bioinformatics analysis

Gene Runner (Gene Runner 6.5.50) was used to design the primers. The sequencing results were analyzed by using BioEdit (BioEdit 7.2.1). The identified nucleotide variant was studied through the UCSC Genome Browser (https://genome.ucsc.edu) and ClinVar (www.ncbi.nlm.nih.gov/clinvar) and Iranome (http://www.iranome.ir/)databases.

## Results

### Clinical information

**Fig. 1:**
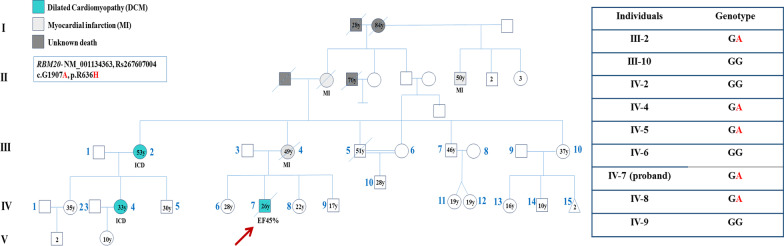
The pedigree of the index family with hereditary dilated cardiomyopathy is illustrated herein. The green-filled square and circle indicate affected males and females, respectively. The gray squares and circles with diagonal lines indicate the deceased males and females, respectively. A thick red arrow in the pedigree specifies the proband. For the pathogenic nucleotide variation in *RBM20*, c.G1907A, the wild type allele is shown by G, and the potentially pathogenic variant is indicated by A. The genotypes pinpoint the co-segregation of c.G1907A (p.R636H) in the heterozygous form (GA) in all the affected members (III-2, IV-4, and IV-7), whereas the 2 unaffected family members (III-10 and IV-2) were wild type homozygous states (GG)

The family of interest comprised a 2-generation pedigree with DCM (Fig. 1). A positive family history of DCM, myocardial infarction, and sudden cardiac death was reported from the maternal side of the family.

The proband in the family (IV-7) was a 26-year-old man who presented initially with retrosternal chest pain, a slightly elevated serum troponin level, and mild left ventricular systolic dysfunction. He denied any palpitation, lightheadedness, syncope, shortness of breath, or weakness. His past medical history was also unremarkable. Of note, the proband’s mother (III-4) and maternal uncle (III-5) had passed away (sudden death), and his maternal aunt was a known case of nonischemic DCM.

One of the proband’s cousins, a 33-year-old woman (IV-4), was also under medical care because of frequent premature ventricular ectopy and mildly reduced left ventricular systolic function. Electrocardiography showed normal sinus rhythm with normal QRS morphology, normal QT intervals, and no pathologic ST-segment or T-wave changes.

Transthoracic echocardiography revealed a normal left ventricular size, mildly reduced systolic function (ejection fraction: 45–50%), and no significant valve problems. Additionally, diastolic function and pulmonary artery pressure were normal. The patient also had reduced longitudinal strain, especially in the basal myocardial segments, with a global longitudinal strain value of − 6.1%.

Cardiac magnetic resonance imaging confirmed the mildly reduced systolic function (ejection fraction: 44%) and showed a left ventricular end-diastolic volume index of 121 mL/m^2^. The right ventricle was mildly enlarged, with a right ventricular end-diastolic volume index of 113 mL/m^2^ and a right ventricular ejection fraction of 40%. Tissue characterization revealed no remarkable myocardial edema or fibrosis.

A 24-h Holter monitoring test was negative for any sustained supra- or ventricular arrhythmia.

With the impression of familial DCM, the patient was started on a goal-directed medical treatment, including the administration of bisoprolol, spironolactone, and sacubitril/valsartan. Empagliflozin was added to the medications subsequently. He was also counseled concerning the implantation of a cardioverter-defibrillator considering his high-risk family history (sudden death in the mother and the uncle); however, he refused to undergo the procedure. He was compliant with his medications for 6 months and remained stable clinically with no symptoms of chest pain, shortness of breath, palpitation, or syncope. Still, he refused to continue with the goal-directed medical treatment. (He found the treatment futile and avoided medical follow-up for fear of the ongoing COVID-19 pandemic.) Eleven months after the diagnosis, he sought medical care because of coffee-ground emesis, which was evaluated via esophagogastroduodenoscopy. A course of pantoprazole was prescribed. Two months later and in a physical altercation, the patient suddenly collapsed and passed away.

The other enrolled family members (III-10, IV-b2, IV-5, IV-6, IV-8, and IV-9) were apparently normal. Screening echocardiography was performed on all these individuals and showed a borderline ejection fraction (50%–55%) and a mildly reduced global longitudinal strain value (− 19.7%) in the 22-year-old sister of the patient (IV-8).

### Genetic finding

An unbiased next-generation DNA sequencing, encompassing the entire coding exons (WES), was carried out to scrutinize the causative genetic variation in the family. WES was accomplished with a mean target coverage rate of 100 × . The results were consecutively analyzed with Bowtie [19], FreeBayes [20], and SnpSift. Regarding to databases filtering and pathogenicity evaluation, three variants were chosen for further analysis.

**Fig. 2:**
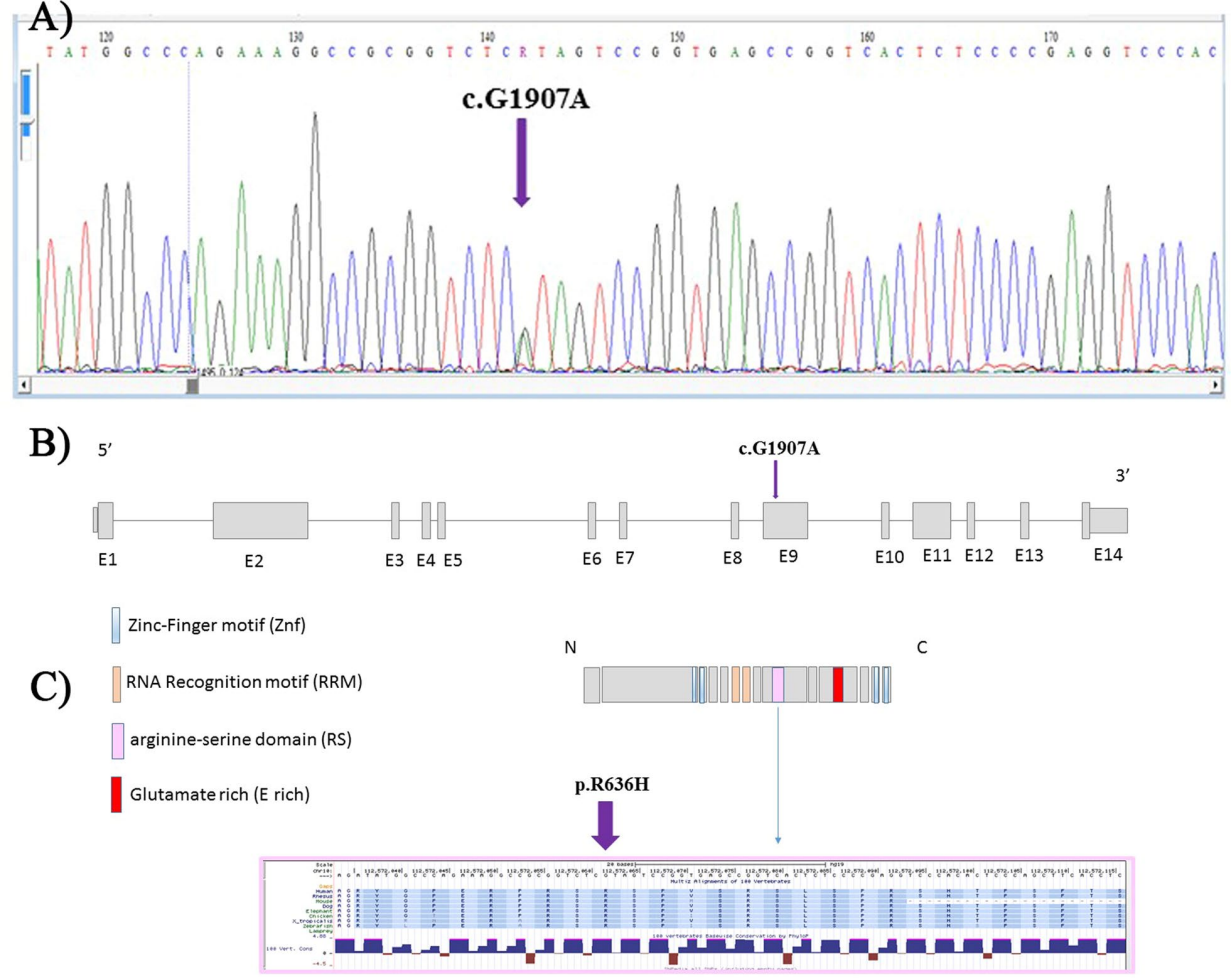
The image depicts the identification of a *RBM20* nucleotide change in a family with dilated cardiomyopathy. **A** The image shows the confirmation of the variant by Sanger sequencing. **B** and **C** The images demonstrate the genomic organization of the human *RBM20* gene and present a schematic representation of the RBM20 protein with the predicted functional domains, and amino acid alignment of the RBM20 arginine-serine–rich domain among vertebrate

The WES outcome revealed a missense pathogenic/likely pathogenic variant, c.1907G > A (rs267607004, NM_0011343), in the *RBM20* gene in the index patient (Fig. 2) along with two other nucleotide variations (Additional file 1: Table S1). The c.1907G > A transition was harbored in a very highly conserved part of exon 9 of the *RBM20* gene located on Chr10: 112,572,062 (GRCh37/hg19), resulting in an Arg636-to-His substitution in the arginine-serine (RS) domain (Fig. 2). This variant is reported neither in the 1000 Genomes Project nor in Iranome databases.

The in silico analysis of this variant through CADD (25.2), PolyPhen-2 (D), SIFT (D), PROVEAN (D), and MutationTaster (D) predicted that it would be damaging. This variant also met the ACMG criteria (PM1, PM2, PM5, PP3, and PP5) and was itemized as a pathogenic variant.

Sanger sequencing of this variant was performed in the proband and all 8 available family members in order to confirm the presence and the pattern of inheritance. The Sanger sequencing upshots demonstrated that the index patient (IV-7) and 1 of his aunts (III-2, who had an implantable cardioverter-defibrillator) carried the pathogenic variant in a heterozygous status (GA), while the other unaffected aunt (III-10) did not carry this pathogenic variant (GG) (Fig. 1).

The proband’s brother (IV-9) and older sister (IV-6) were a wild type for the variant. In addition, his younger sister (IV-8) also carried the variant in a heterozygous form and presented with a borderline left ventricular ejection fraction (50%–55%) and a mildly reduced global longitudinal strain value (− 19.7%) in her echocardiographic examination.

The proband’s aunt (III-2) had 3 children. Two of them (1 boy [IV-5] and 1 girl [IV-4]) were genetically heterozygous for the *RBM20* variant (GA), while the other daughter (IV-2) was a wild type for the variant (Fig. 1). The proband’s male first cousin (IV-5), 30 years of age, was a carrier of the pathogenic variant, but he exhibited no DCM symptoms. In contrast, the proband’s female first cousin (IV-4), 33 years old, was a carrier of the pathogenic variant and needed an implantable cardioverter-defibrillator due to ventricular arrhythmias.

## Discussion

DCM can be caused by mutations in genes that mostly encode the cytoskeletal, sarcomeric, or contractile proteins of myocytes [6, 21, 22]. These genes are almost directly involved in the generation and/or transmission of contractile force via protein–protein interactions. In addition, pathogenic mutations in unsuspected DCM genes like *LMNA* and *EYA4* lead to DCM as well [23, 24]. Nucleotide transitions in Lamin A/C, encoded by *LMNA* with unknown mechanisms, cause DCM and conduction system disorders [23–25]. Further mutations in *EYA4*, a transcriptional coactivator, could cause syndromic DCM with sensorineural hearing loss [24]. Accumulating evidence indicates that alternative splicing perturbations contribute to cardiac diseases. Some splicing factors, including *RBM20, RBM24, Rbfox*, and *SF3B1*, play critical roles in developing adult cardiac tissue [26–29]. In 2009, *RBM20* was introduced as a familial DCM gene [30]. More recently, *RBM20* nucleotide variation have been found in familial and sporadic DCM cases [31]. Due to Iranome database 57 exonic nucleotide variants of *RBM20* gene (Additional file 2: Table S2) were reported in healthy Iranian population. Additionally, 106 and 2 nucleotide variations were announced in introns and 3’-UTR of *RBM20* gene.

In the current investigation, with the aid of WES, we succeeded in identifying a pathogenic variant, c.1907G > A (rs26760704), in the *RBM20* gene in the members of an Iranian family with DCM. To the best of our knowledge, this is the first report of a pathogenic nucleotide variant in the *RBM20* gene in Iranian patients with DCM.

The *RBM20* gene, composed of 14 exons, encodes the RNA-binding motif protein of 1227 amino acid residues in several conserved domains, including -a arginine/serine (RS)-rich region just following the prototype RNA recognistion motif (RRM) domain (Fig. 2) [26, 32, 33]. The *RBM20* gene has an extremely high expression in both atria and ventricles [34].

**Fig. 3:**
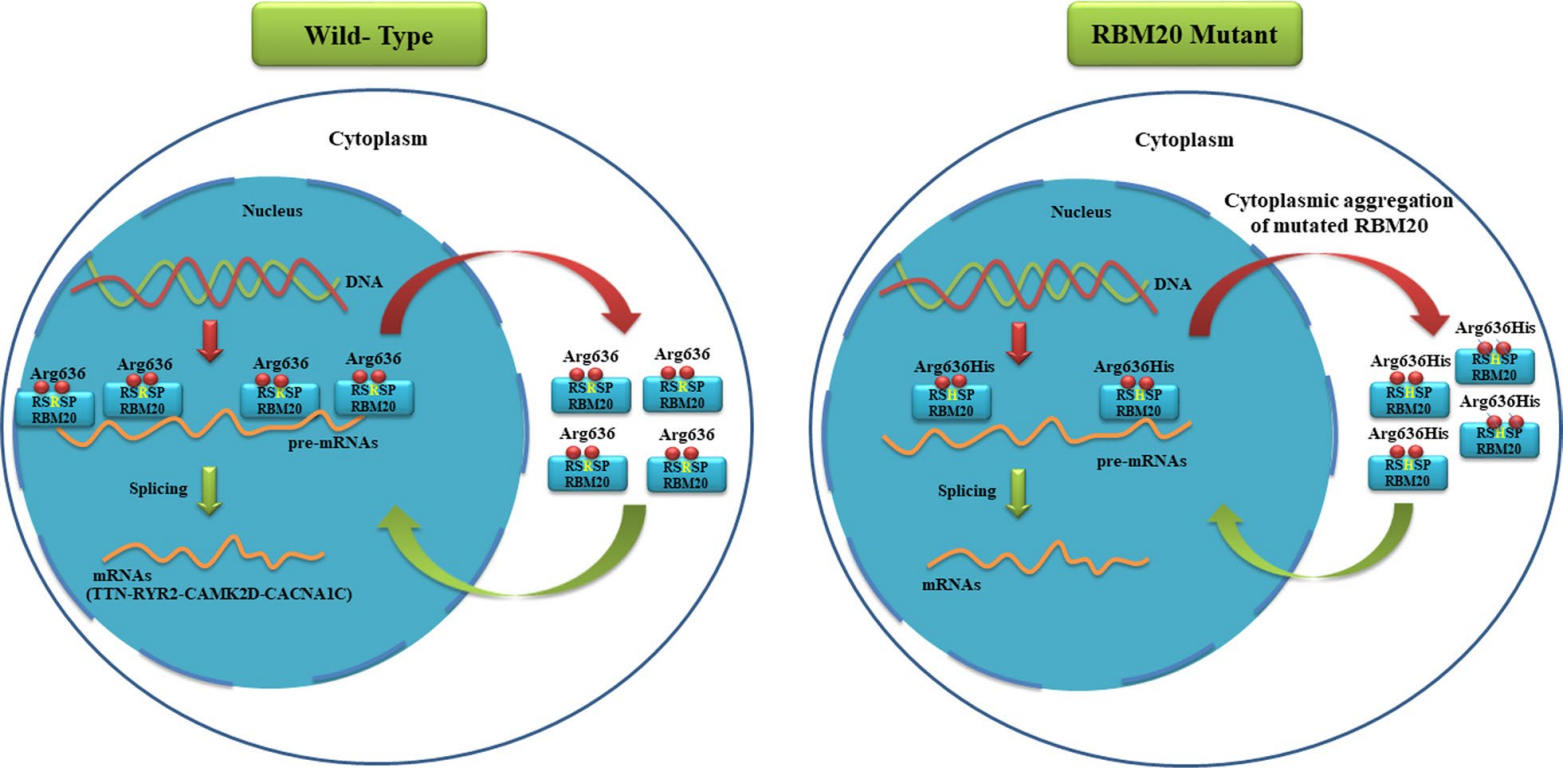
The image shows the mislocalization of RBM20 due to a pathologic nucleotide variation in the RS domain. In the wild-type form, all RBM20 proteins are transferred from the cytoplasm to the nucleus after phosphorylation. In contrast, in the mutant form, phosphorylation is abrupt, and most RBM20 proteins aggregate in the cytoplasm

RBM20 is a key heart splice regulator that controls the process of some important transcripts. It exists in considerable amounts in striated muscles, with the upper level of expression in cardiac tissue. [35] Previous studies have demonstrated the mislocalization of RBM20 due to the aberrant phosphorylation of the RSRSP domain. [33, 36] Therefore, patients with heterozygous forms of mutations in the RS domain have lower concentrations of RBM20 in the nucleus, leading to functional deficiencies of RBM20 (Fig. 3). [36] The mis-splicing of some targets of RBM20 in consequence of mutations in the *RBM20* gene results in progressive DCM with conduction diseases, including atrial and ventricular arrhythmias. Although the exact mechanism of RBM20 remains to be investigated, numerous pathogenic nucleotide transition variants, in conjunction with a mutational hot spot in an RS-rich domain, have been reported to be the causes of DCM. [31]

Pathogenic nucleotide variations in the RS domain result in the disruption of binding with other splicing factors. Hence, these mutations have the potential to impair the normal processing of the splicing of transcripts such as *Titin*, culminating in highly penetrant DCM. [37, 38]

A hot-spot region in RBM20 includes an arginine-serine-arginine-serine-proline (RSRSP) stretch in 634 to 638 amino acids in the RS-rich region domain. Most RBM20 mutations harbored in the RS-rich region are heterozygous missense loss- or reduction-of-function mutations. [30, 31, 39, 40] Earlier investigations demonstrated R634Q, R634W, S635A, R636S, R636H, R636C, S637G, and P638L in familial and sporadic DCM patients [26, 30, 33, 39, 41–43]. The R636H mutation has been reported in familial and sporadic forms of DCM in several studies carried out in different countries (Table 1) [30, 39–41, 43–50]. Subsequent research on patients carrying the RBM20 mutations confirmed that DCM symptoms manifested themselves at an early age, followed by a high degree of morbidity and mortality even in young children due to the deterioration of cardiac systolic function toward end-stage heart failure [30, 51, 52].

**Table 1:** All reported pathogenic nucleotide variations in the RS-region domain of RBM20

Mutation	Type	Country	References
R634Q R636S R636H S637G P638L	Hetero	USA	[30]
R634Q R634W R636C R636H	Hetero	USA	[39]
S637G	Hetero	Germany	[42]
R636C	Hetero	USA	[53]
P638L R634W R636C R634Q R636H	Hetero	USA	[40]
S635A	Hetero	Germany	[26]
R636H	Hetero	USA	[43]
R636H	Hetero	Canada	[41]
R636H	Hetero	Finland	[44]
R634Q R634W S635A R636S R636H R636C S637G P638L	Hetero	Germany	[49]
R634W	Hetero	Tokyo	[33]
R636H	Hetero	Spain	[45]
R636H R634Q R636S P638L	Hetero	Denmark	[46]
R634Q R636S R636H	Hetero	Denmark	[47]
R636H	Hetero	China	[50]
P638L	Hetero	Germany	[36]
R636H	Hetero	Vietnam	[48]
R634Q R634W	Hetero	Greece	[54]

In line with the aforementioned studies, we found an R636H mutation in a hot-spot domain (the RS-region domain) running in a dominant pattern in a family with familial DCM. The proband (the index patient), as well as his sister, aunt, and cousins (the affected aunt’s children), carried the mutant allele in a heterozygous form. Except for a cousin with a c.1907G > A variation, almost all the carriers showed the DCM phenotype. The other family members with the wild type of nucleotide variation were clinically normal, as expected. Unfortunately, the index patient, together with his mother and one of his uncles, passed away due to sudden cardiac death.

Although, previously reported confirmed that patients carrying the RBM20 mutations in RS domain showed the DCM phenotype at an early age with high penetrance along morbidity and mortality. Phenotype heterogeneity in the family specially the severe phenotype of the proband might be due to other heterozygous variants found in TTN (P.G25367S) and RYR1 (p.4265_4268del) which are not co-segregated in other available family members along with the existed pathogenic R636H variant in RBM20. Further experimental studies are needed to clarify the association and the impact of these variants on DCM.

Recently, more than 100 genes have been reported in association with DCM [55, 56]. There is, however, a dearth of information on the genetics of DCM in Iranian patients. Previous investigations have demonstrated associations between DCM and *δSG* [57]*,* *LMNA* [58]*,* *FLNC* [59]*,* desmoplakin (DSP) [60], *MYBPC3*, troponin T type 2 (*TNNT2*), myosin heavy polypeptide 7 (*MYH7*) [61], and *PLN* [62]*.* The finding of a mutation in the *RBM20* gene in the present study, along with other studies, in Iranian patients highlights the genetic heterogeneity and wide spectrum of mutations in Iranians and underscores the need for further comprehensive studies.

## Supplementary Information

**Additional file 1.**The other variants identified in an index patient.

**Additional file 2.**All reported exonic nucleotide variations in *RBM20* gene in Iranome database.

## Data Availability

The accession number of the reported variant in paper is available with the following accession number; rs267607004, NM_0011343.The data sets presented in the present study can be accessed in online repositories. The identified nucleotide transitions were investigated through the UCSC Genome Browser (https://genome.ucsc.edu) and ClinVar (www.ncbi.nlm.nih.gov/clinvar) databases. In silico predictive software tools entailing Combined Annotation Dependent Depletion (CADD Phred > 15) (https://cadd.gs.washington.edu/), SIFT (https://sift.bii.a-star.edu.sg), PROVEAN (provean.jcvi.org), PolyPhen-2 (genetics.bwh.harvard.edu/pph2), and MutationTaster (www.mutationtaster.org) were employed to study the pathogenesis of the detected nucleotide variation. The accession number and all the repositories used for the study are both mentioned in the article and declarations part as well.
