# Supplementary material for: Dilated cardiomyopathy caused by a pathogenic nucleotide variant in RBM20 in an Iranian family
Source: BMC Med Genomics. 2022 May 8;15:106. doi: 10.1186/s12920-022-01262-4 (PMC9079971; doi:10.1186/s12920-022-01262-4)
Supplement: Supplementary file 1 — Additional file 1.The other variants identified in an index patient. [file 12920_2022_1262_MOESM1_ESM.docx]

|  |  |  |  |  |
| --- | --- | --- | --- | --- |

**Supplementary Table 1:** The other variants identified in an index patient.

| **Gene** | **NM/NP** | **Variant** | **Type** | **RS** | **Location** | **In silico assessments** | | |
| --- | --- | --- | --- | --- | --- | --- | --- | --- |
|  |  |  |  |  |  | **Mutation taster** | **Provean** | **Sift** |
| TTN | NM_003319 | c.G76099A  p.G25367S | Het | Novel | chr2:179398048C> | disease causing | Deleterious | Damaging |
| RYR1 | NM_001042723 | c.12795_12803delGGGCGCGGA  p.4265_4268del | Het | rs759582696 | chr19:39055784_39055792 | polymorphism | - | - |
