# Supplementary material for: Dilated cardiomyopathy caused by a pathogenic nucleotide variant in RBM20 in an Iranian family
Source: BMC Med Genomics. 2022 May 8;15:106. doi: 10.1186/s12920-022-01262-4 (PMC9079971; doi:10.1186/s12920-022-01262-4)
Supplement: Supplementary file 2 — Additional file 2.All reported exonic nucleotide variations in RBM20 gene in Iranome database. [file 12920_2022_1262_MOESM2_ESM.docx]

**Supplementary Table 2**

All reported exonic nucleotide variations in *RBM20* gene in Iranome database.

| Number | Chromosome:Variant | dbSNP | Protein Consequence | Transcript Consequence | Annotation | Exon Number | Allele Frequency |
| --- | --- | --- | --- | --- | --- | --- | --- |
| 1 | 10:112404292 C / G |  | p.Pro27Arg | c.80C>G | **missense** | 1 | 0.001276 |
| 2 | 10:112404302 G / A | rs35141404 | p.Arg30= | c.90G>A | **synonymous** | 1 | 0.1255 |
| 3 | 10:112404320 C / T |  | p.Ser36= | c.108C>T | **synonymous** | 1 | 0.000641 |
| 4 | 10:112404333 A / C |  | p.Met41Leu | c.121A>C | **missense** | 1 | 0.000642 |
| 5 | 10:112404335 _ / GCA | rs528952546  rs397516593 | p.Gln43_Pro44insGln | c.122_123insGCA | **disruptive inframe insertion** | 1 | 0.006443 |
| 6 | 10:112540786 C / T |  | p.Pro140Leu | c.419C>T | **missense** | 2 | 0.000625 |
| 7 | 10:112540787 G / A | rs776692347 | p.Pro140= | c.420G>A | **synonymous** | 2 | 0.000625 |
| 8 | 10:112540884 C / A | rs7908490 | p.Pro173Thr | c.517C>A | **missense** | 2 | 0.004375 |
| 9 | 10:112540896 A / T | rs397516621 | p.Thr177Ser | c.529A>T | **missense** | 2 | 0.000625 |
| 10 | 10:112541047 G / T | rs202238753 | p.Gly227Val | c.680G>T | **missense** | 2 | 0.00875 |
| 11 | 10:112541062 G / A | rs61735268 | p.Gly232Asp | c.695G>A | **missense** | 2 | 0.003125 |
| 12 | 10:112541143 G / T |  | p.Gly259Val | c.776G>T | **missense** | 2 | 0.000625 |
| 13 | 10:112541210 T / C |  | p.Asp281= | c.843T>C | **synonymous** | 2 | 0.000625 |
| 14 | 10:112541344 C / T | rs754268847 | p.Ser326Leu | c.977C>T | **missense** | 2 | 0.000625 |
| 15 | 10:112541407 A / G | rs794729142 | p.Tyr347Cys | c.1040A>G | **missense** | 2 | 0.000625 |
| 16 | 10:112541445 A / G |  | p.Thr360Ala | c.1078A>G | **missense** | 2 | 0.000625 |
| 17 | 10:112541524 A / G |  | p.Gln386Arg | c.1157A>G | **missense** | 2 | 0.000625 |
| 18 | 10:112541538 G / A | rs763101142 | p.Val391Met | c.1171G>A | **missense** | 2 | 0.00375 |
| 19 | 10:112541585 C / T |  | p.Ala406= | c.1218C>T | **synonymous** | 2 | 0.000625 |
| 20 | 10:112543156 C / T | rs189600634 | p.His436= | c.1308C>T | **synonymous** | 3 | 0.000625 |
| 21 | 10:112544109 C / T |  | p.Arg450Trp | c.1348C>T | **missense** | 4 | 0.000625 |
| 22 | 10:112544139 T / C | rs374014662 | p.Leu460= | c.1378T>C | **synonymous** | 4 | 0.000625 |
| 23 | 10:112544151 C / T |  | p.Pro464Ser | c.1390C>T | **missense** | 4 | 0.00125 |
| 24 | 10:112544575 A / G | rs751559376 | p.Ser485= | c.1455A>G | **synonymous** | 5 | 0.00125 |
| 25 | 10:112544655 C / T | rs7077757 |  | c.1527+8C>T | **splice region** | 5 | 0.09938 |
| 26 | 10:112557355 G / T | rs761674598 | p.Gly539= | c.1617G>T | **synonymous** | 6 | 0.001875 |
| 27 | 10:112557397 G / A | rs375626512 | p.Ser553= | c.1659G>A | **synonymous** | 6 | 0.000625 |
| 28 | 10:112559572 G / A |  | p.Ala566Thr | c.1696G>A | **missense** | 7 | 0.003125 |
| 29 | 10:112559578 C / G |  | p.Gln568Glu | c.1702C>G | **missense** | 7 | 0.000625 |
| 30 | 10:112570179 C / T |  | p.Ile613= | c.1839C>T | **synonymous** | 8 | 0.000625 |
| 31 | 10:112570195 A / T |  | p.Arg619Trp | c.1855A>T | **missense** | 8 | 0.000625 |
| 32 | 10:112572069 G / A | rs74339620 | p.Pro638= | c.1914G>A | **synonymous** | 9 | 0.003125 |
| 33 | 10:112572114 C / A |  | p.Thr653= | c.1959C>A | **synonymous** | 9 | 0.000625 |
| 34 | 10:112572140 C / T | rs752484044 | p.Pro662Leu | c.1985C>T | **missense** | 9 | 0.000625 |
| 35 | 10:112572147 C / T | rs41292592 | p.Pro664= | c.1992C>T | **synonymous** | 9 | 0.000625 |
| 36 | 10:112572328 G / A | rs190416018 | p.Glu725Lys | c.2173G>A | **missense** | 9 | 0.000625 |
| 37 | 10:112572368 C / T | rs397516601 | p.Pro738Leu | c.2213C>T | **missense** | 9 | 0.000625 |
| 38 | 10:112572458 G / C | rs1417635 | p.Trp768Ser | c.2303G>C | **missense** | 9 | 1 |
| 39 | 10:112572458 G / T | rs1417635 | p.Trp768Leu | c.2303G>T | **missense** | 9 | 1 |
| 40 | 10:112572473 A / G | rs181769913 | p.Lys773Arg | c.2318A>G | **missense** | 9 | 0.000625 |
| 41 | 10:112572513 C / T |  | p.Asp786= | c.2358C>T | **synonymous** | 9 | 0.000625 |
| 42 | 10:112572548 C / T | rs730880184 | p.Pro798Leu | c.2393C>T | **missense** | 9 | 0.000625 |
| 43 | 10:112579912 C / T | rs368291152 | p.Pro878Leu | c.2633C>T | **missense** | 10 | 0.000625 |
| 44 | 10:112581138 A / G | rs397516608 | p.Ile921Val | c.2761A>G | **missense** | 11 | 0.000625 |
| 45 | 10:112581220 G / A | rs763189178 | p.Cys948Tyr | c.2843G>A | **missense** | 11 | 0.000625 |
| 46 | 10:112581239 C / T | rs751775695 | p.Asp954= | c.2862C>T | **synonymous** | 11 | 0.001875 |
| 47 | 10:112581453 G / A | rs866975469 | p.Glu1026Lys | c.3076G>A | **missense** | 11 | 0.000625 |
| 48 | 10:112581521 C / T | rs75858380 | p.Ser1048= | c.3144C>T | **synonymous** | 11 | 0.003125 |
| 49 | 10:112581547 G / A | rs188054898 | p.Arg1057Gln | c.3170G>A | **missense** | 11 | 0.01125 |
| 50 | 10:112581642 C / A | rs147356378 | p.Pro1089Thr | c.3265C>A | **missense** | 11 | 0.00125 |
| 51 | 10:112583294 G / A | rs116908219 | p.Glu1125Lys | c.3373G>A | **missense** | 12 | 0.00375 |
| 52 | 10:112590882 G / A |  | p.Ser1172Asn | c.3515G>A | **missense** | 13 | 0.000625 |
| 53 | 10:112590912 G / A | rs563762318 | p.Arg1182His | c.3545G>A | **missense** | 13 | 0.000625 |
| 54 | 10:112595656 C / T |  | p.Leu1202Phe | c.3604C>T | **missense** | 14 | 0.000625 |
| 55 | 10:112595685 G / A | rs781373863 | p.Pro1211= | c.3633G>A | **synonymous** | 14 | 0.000625 |
| 56 | 10:112595700 C / T | rs554167951 | p.Ser1216= | c.3648C>T | **synonymous** | 14 | 0.000626 |
| 57 | 10:112595719 G / C | rs942077 | p.Glu1223Gln | c.3667G>C | **missense** | 14 | 0.7672 |
